# Comparative Repeatomics Uncovers Genome Diversification in Gasteropelecidae Fishes

**DOI:** 10.3390/ijms27198720

**Published:** 2026-09-29

**Authors:** Pablo Rhuan Silva Veiga, Jhon Alex Dziechciarz Vidal, Thomas Liehr, Marcelo de Bello Cioffi

**Affiliations:** 1 Departamento de Genética e Evolução, Universidade Federal de São Carlos, São Carlos 13565-905, SP, Brazil; pablo.veiga@estudante.ufscar.br (P.R.S.V.); jhonalex279@gmail.com (J.A.D.V.); 2 Institute of Human Genetics, Jena University Hospital, Friedrich Schiller University, 07747 Jena, Germany

**Keywords:** satellite DNAs, sex chromosome evolution, ZZ/ZW system, transposable elements, chromosomal evolution

## Abstract

Repetitive DNA contributes to genome and chromosome evolution, yet its diversification remains poorly characterized by Neotropical fishes. Gasteropelecidae provides an informative system because its three genera share an ancient ZZ/ZW sex chromosome system despite lineage-specific W chromosome differentiation. Here, we characterized and compared the repeatomes of three Gasteropelecidae species to investigate how repetitive DNA diversification relates to genome and chromosome evolution. We analyzed *Carnegiella strigata, Gasteropelecus levis, and Thoracocharax stellatus*, with *Triportheus auritus* as a comparative lineage. Despite ~30–40 Myr of divergence, Gasteropelecidae species showed remarkably conserved repeatome composition, with repetitive DNA accounting for ~25–32% of their genomes and consistently dominated by retroelements. In contrast, *T. auritus* displayed a distinct repeatome composition, with DNA transposons representing the most abundant annotated repeat class. Satellitomes were more dynamic: 62% of the 235 identified satDNA families showed homology across taxa, consistent with differential amplification of shared satellite sequences. *G. levis* exhibited 19 female-biased satDNA families, whereas FISH mapping in *T. stellatus* provided direct cytogenetic evidence of W chromosome enrichment for specific satDNAs. Thus, conserved genome-wide repeat landscapes coexist with rapid satellitome diversification, with lineage-specific satDNA amplification potentially contributing to W chromosome differentiation while the ancestral ZZ/ZW system remains conserved.

## 1. Introduction

Formerly, repetitive DNA sequences were denominated as “junk DNA” because they lack protein-coding potential and any apparent biological function [1]. In recent decades, this view has substantially changed, as they are now considered an integral component of eukaryotic genomes with key roles in chromosome architecture, genome stability, gene regulation, and evolutionary innovations [2]. Repetitive DNA is a major determinant of genome sizes, accounting for much of their variation across species [3,4], and is classified into two main categories: (i) dispersed repeats, which include Transposable Elements (TEs) (LINEs, SINEs, and LTR elements) and DNA transposons, and (ii) tandem repeats, which include satellite DNAs (satDNAs), minisatellites, and microsatellites [2,3]. These sequences play important roles in establishing chromatin states, promoting heterochromatin formation, regulating gene expression, and mediating epigenetic pathways, including small RNA-dependent silencing mechanisms [5,6]. Furthermore, they are important in chromosome inheritance, as satellite DNAs form the structural foundation of the centromeric and pericentromeric regions, which are essential for kinetochore assembly, chromosome segregation, and centromere identity [7,8].

The advent of high-throughput sequencing has made it possible to characterize entire repeatomes genome-wide, giving insight into the diversity, abundance, and evolutionary dynamics of TEs and tandem repeats [9,10,11,12]. Lineage-specific changes in the abundance and the chromosomal distribution of repetitive elements are also recognized as major drivers of genome and chromosomal evolution [13,14,15,16]. The analysis of a complete set of satDNAs (satellitome) [11] has emerged as a powerful approach for studying chromosome evolution, revealing patterns of satDNA amplification and turnover [17,18], the fast evolution of centromeric repeats [19,20], and genome-wide turnover of repetitive DNAs involved in sex chromosome differentiation [21,22,23,24].

**Figure 1:**
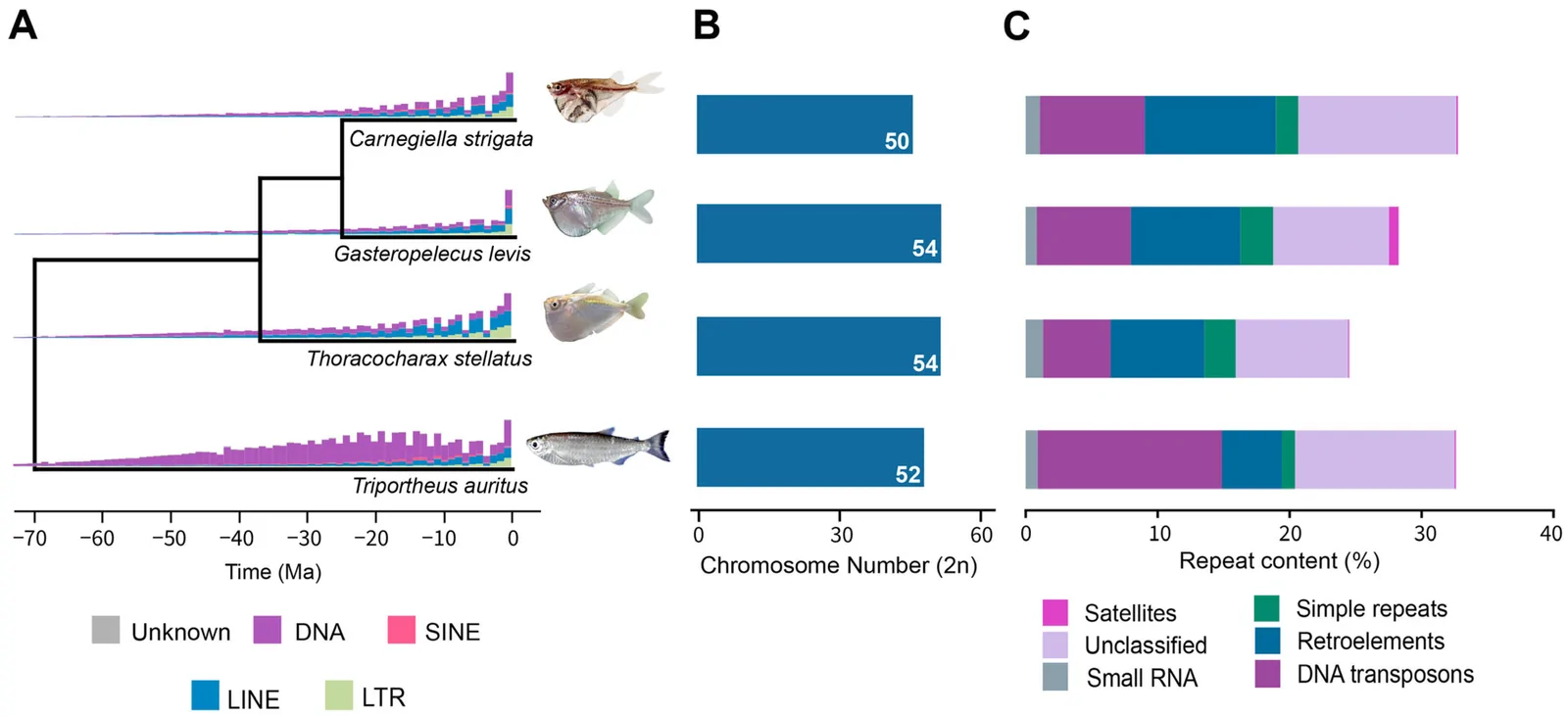
Evolutionary relationships, chromosome numbers, and genomic repeat content in Gasteropelecidae and the outgroup *Triportheus auritus.* (**A**) Phylogenetic relationships and divergence times are reproduced from Melo et al. [36]. The repeat landscapes along the branches show the relative abundance of major transposable element classes, including DNA transposons, SINEs, LINEs, and LTR retrotransposons. (**B**) The diploid numbers (2n) of each species are displayed, along with their repeat content (**C**).

Although repeatome analyses (i.e., the identification and characterization of repetitive DNA sequences across a genome) have expanded rapidly, comprehensive studies integrating genomic and cytogenetic data remain scarce for many vertebrate groups. Such a gap is particularly evident in Neotropical fishes, where the extraordinary chromosomal diversity observed in this group (as revised in [25]) contrasts with the limited availability of genomic resources and datasets. Among Neotropical fishes, Gasteropelecidae (Characiformes) is an excellent model for investigating the relationship between repetitive DNA, karyotype, and sex chromosome evolution. These small Neotropical fishes, also known as freshwater “hatchetfishes” or “butterfly fishes,” encompass nine valid species spanning three genera: *Carnegiella, Gasteropelecus,* and *Thoracocharax* [26]. They occur in the Amazon, Orinoco, Paraguay, and Maroni river basins, demonstrating extensive distribution throughout Panama and all South American countries, except Chile [27]. These species exhibit moderate interspecific variation in diploid chromosome number (2n), with *C. strigata* having 2n = 50 chromosomes, whereas *G. levis* and *T. stellatus* possess 2n = 54 [28,29,30]. Despite this variation, the three species share a broadly similar chromosomal organization, with predominantly acrocentric chromosomes and a highly differentiated ZZ/ZW sex chromosome system. Also, in all three species, the Z chromosome is the largest element of the karyotype, whereas the W chromosome is considerably smaller, although its morphology varies among species [28]. Fish sex-chromosome systems span a broad range of evolutionary ages, from relatively recent systems to deeply conserved systems [31,32,33,34]. In Gasteropelecidae and Triportheidae, the homologous ZZ/ZW sex chromosome system is associated with lineages that diverged at ~70 Mya [28,35,36] (Figure 1).

Notwithstanding this extraordinary evolutionary stability, the W chromosome has undergone distinct lineage-specific evolutionary pathways, characterized by significant structural alterations and varied accumulation of repeats, resulting in notable interspecific differences in size and form [28]. Recent satellitome investigations in Triportheidae showed that W-chromosome differentiation was primarily driven by the autonomous amplification of lineage-specific satDNA groups, with only a limited number of satellites retained across species [37,38]. These results suggest that rapid turnover of repetitive DNAs can occur in a conserved homologous sex chromosome system. However, the genomic architecture underlying this diversification is poorly understood, especially in Gasteropelecidae, whose repeatome has not been characterized for any species.

Here, we present the first comparative characterization of the repetitive DNA landscape in Gasteropelecidae by integrating genome-wide repeatome analyses with molecular cytogenetics. We characterized the repeatome with a focus on the satellitome composition of three representative species, named *Thoracocharax stellatus*, *Gasteropelecus levis*, and *Carnegiella strigata*, encompassing all three extant genera of the family. We compared the diversity and abundance of repetitive DNA families across species to investigate patterns of repeat evolution and genome organization. In addition, we validated the in situ chromosomal distribution of selected satDNA families in *T. stellatus*, providing a cytogenetic framework for interpreting genome-wide repeatome data. By combining these complementary approaches, we evaluate how repetitive DNA contributes to genome architecture, chromosome evolution, and the differentiation of the ancient ZZ/ZW sex chromosome system in Gasteropelecidae.

## 2. Results

### 2.1. Characterization and Comparison of Repeatomes

Repeatome profiling of three representative Gasteropelecidae species showed that their repetitive DNA landscapes are highly conserved (Figure 1, Table 1). The total repetitive DNA accounted for ~24–32% of the genomes, with retroelements representing the largest fraction of annotated repeats (~7–9%), particularly Long Interspersed Nuclear Elements (LINEs) and Long Terminal Repeat (LTR) retrotransposons, exceeding the contribution of DNA transposons (5–7%). Consequently, interspersed repeats comprised 13–17% of the genomes. LINEs were the most abundant class of retroelements, occupying ~6% of the genome, while LTR retrotransposons accounted for 1.4–2.5% SINEs, Helitrons, satDNAs, rRNA, low-complexity sequences, and other repeats were relatively low in abundance, typically comprising less than 1% of the genome. DNA transposons were found in similar proportions in all three species, between 5 and 7% (Figure 1, Table 1). Conversely, the total repeat content (32.60%) of the outgroup *T. auritus* was similar to that of Gasteropelecidae. However, its repeatome composition was significantly different, with DNA transposons being the most abundant annotated repeat class (13.72%), while LINEs (3.06%) and LTR retrotransposons (0.73%) were proportionally less abundant than in Gasteropelecidae (Figure 1, Table 1). These differences should be interpreted as differences in repeatome composition rather than as evidence of a specific ancestral repeatome state.

### 2.2. General Features and Homology of Satellitomes

The satellitomes varied considerably in composition among the three Gasteropelecidae species (Tables S1–S3). *Carnegiella strigata* had the most diverse satellitome, with 92 satDNA families (RUL = 13–2314 bp; A + T content = 23.9–78.3%) accounting for 8.0% of the genome (Table S1), followed by *G. levis*, with 84 families (RUL = 12–3734 bp; A + T = 37.0–76.2%) and a markedly sex-biased total abundance (6.7% in females versus 4.8% in males) (Table S2), and *T. stellatus*, with the smallest repertoire of 59 families (RUL = 17–3094 bp; A + T = 31.5–81.5%) totaling 6.1% of the genome (Table S3). Sequence divergence within families ranged broadly (0.5–21.4% across all species), consistent with satDNA turnover acting heterogeneously across the satellitome rather than uniformly on all repeat lineages. Cross-species comparison (Table 2 and Table S4) revealed that 146 of the 235 satDNA families identified (62%) share detectable homology with at least one family in another taxon, organized into 45 homology groups, while the remaining families appear to be species-specific. Homology was not restricted to the Gasteropelecidae; 12 out of 45 of these groups included satDNAs from the outgroup *T. auritus* (Table S4). Notably, one large homology group (Group 3) included 22 satDNA families from all four taxa, suggesting a shared ancestral satDNA lineage that has since diversified into multiple paralogous families within each genome (Table 2). Overall, 59 of the 279 pairwise comparisons (21%) showed ≥80% sequence identity (Table S4), reflecting strong conservation and likely more recent homogenization events. Comparison of satDNA abundance across the species analyzed (Table S5) showed that most satDNA families were detected at low abundance in multiple species, including species other than those in which they were originally identified. This suggests that the species share a similar repertoire of tandem repeats. However, 55 of the 243 satDNA families were absent from at least one species or sample. *Triportheus auritus* had the highest number of families without a detected match (30).

The comparison of the satellitome between male and female *G. levis* shows a similar repetitive DNA landscape (Table S2). The 84 satDNA families identified in *G. levis* represented overall 6.73% and 4.81% of female and male abundance, respectively (Table S2). Among these families, 19 had a female-biased distribution (F/M ratio > 2.5) and accounted for about 28.6% of the total satDNA fraction recovered from the female library (Table S2). The largest female enhancement was observed for GleSat52 (F/M = 11.10×), GleSat02 (F/M = 4.74×), GleSat71 (F/M = 4.45×), and GleSat05 (F/M = 3.51×). Conversely, only four families showed male-biased enrichment (M/F > 1.5): GleSat16 (M/F = 1.94×), GleSat58 (M/F = 1.98×), GleSat76 (M/F = 1.65×), and GleSat80 (M/F = 1.87×), all with comparatively low abundances. The most abundant satDNA family, GleSat01 (abundance = 1.00% RUL = 216 bp), displayed a remarkably similar distribution between sexes (M/F ≈ 1.06) (Table S2).

### 2.3. Chromosome Mapping of TstSatDNAs

**Figure 2:**
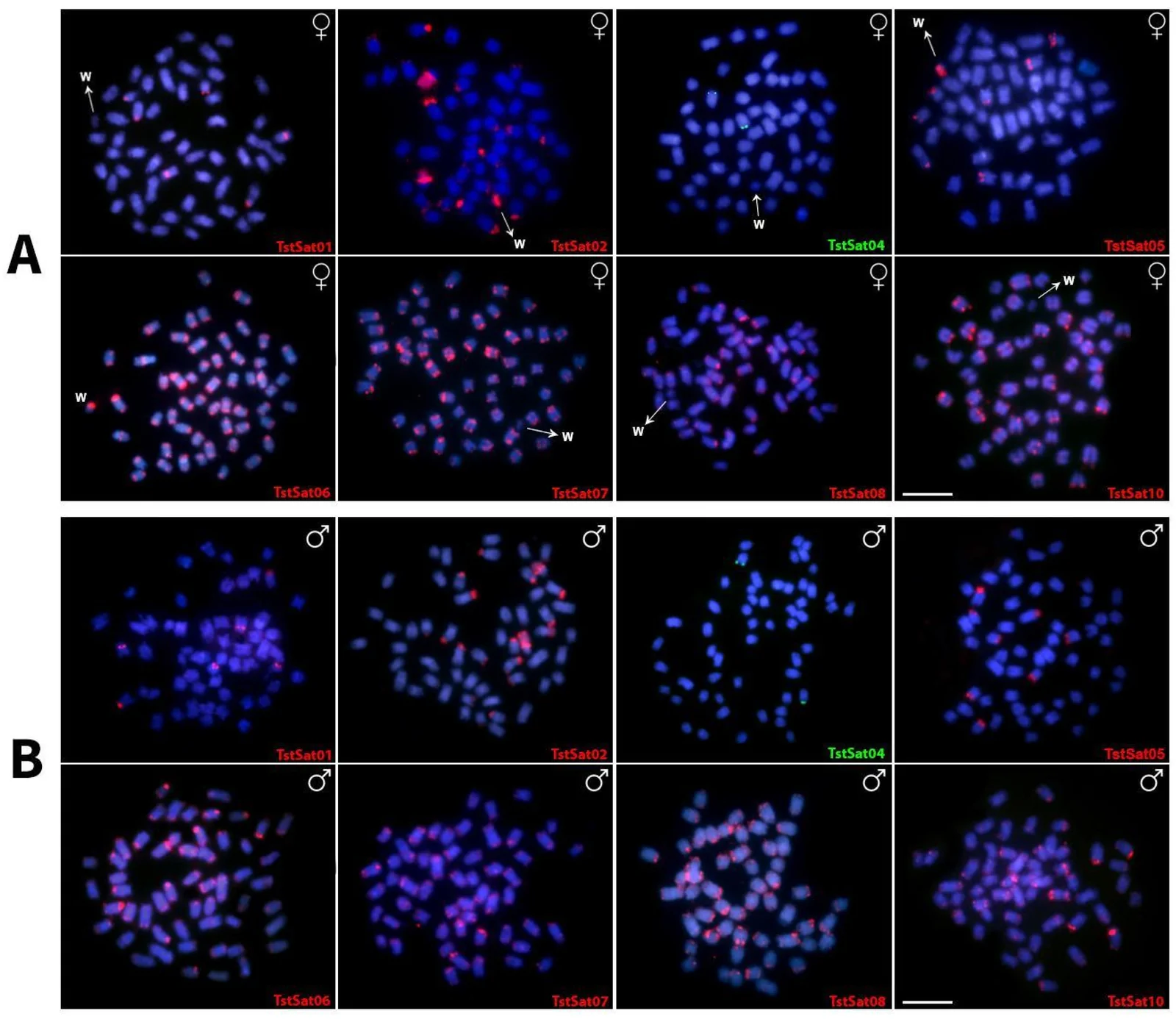
Female (**A**) and male (**B**) metaphase chromosomes of *Thoracocharax stellatus* showing hybridization signals from distinct TstSatDNA families. The names of each satellite DNA family are indicated in the bottom-right corner of each panel in red (Atto-550-dUTP labeled) or green (ATTO-488-dUTP labeled). The W chromosomes are indicated in female metaphases (**A**). Scale bar = 20 µm.

Due to the availability of chromosomal preparations exclusively for *T. stellatus,* fluorescence in situ hybridization (FISH) mapping was restricted to this species. The analyzed satDNA families exhibited strikingly distinct chromosomal distribution patterns (Figure 2). TstSat04 showed a highly localized organization, producing signals on a single chromosome pair, whereas TstSat01 and TstSat05 were restricted to three and four chromosome pairs, respectively. In contrast, TstSat02, TstSat06, TstSat07, TstSat08, and TstSat10 displayed broad chromosomal distributions, with hybridization signals dispersed across numerous chromosome pairs. Notably, TstSat02, TstSat05, and TstSat06 exhibited pronounced accumulation on the W chromosome, indicating preferential amplification within this sex-specific chromosome. TstSat02 also produced a conspicuous signal on a large submetacentric chromosome pair in both males and females (Figure 2).

## 3. Discussion

In Neotropical fishes, lineage-specific bursts of TEs are often linked to genomic diversification, which leads to extensive genome reorganization and changes in repeatome composition after speciation [39,40,41,42]. However, our comparative analyses showed a remarkably conserved overall repeatome composition in Gasteropelecidae species in spite of ~30–40 Mya of evolutionary divergence [36]. Our comparative repeatome analysis yielded a surprisingly conserved repetitive DNA profile across the three Gasteropelecidae genera (Table 1) and, more notably, a consistent hierarchy of repeat classes, with retroelements, particularly LINEs (5.20–5.99%), as the major component, followed by DNA transposons (5.09–7.57%) and LTR retrotransposons (1.03–2.33%) (Table 1 and Tables S1–S3). This pattern contrasts with the more variable repeatome landscapes generally described for other Teleost lineages, in which shifts in TE class dominance are often linked to speciation events [41]. An important aspect of this conserved genomic landscape is the relative stability of chromosome number across Gasteropelecidae, despite considerable structural differentiation of their karyotypes. Although 2n varies only modestly (50–54), numerous pericentric inversions have contributed to changes in chromosome morphology, including structural differentiation of the Z chromosome [28].

To better understand the evolutionary dynamics of the Gasteropelecidae repeatomes, we compared these patterns with a representative species (*T. auritus*) from its sister family, Triportheidae. Despite these lineages diverging at ~ 70 Mya and displaying a homologous ZZ/ZW sex chromosomal system [28], their repeat landscapes differ markedly. Although displaying comparable total repetitive DNA proportions (24–32% of the genome) (Table 1), the contributions of the primary TE categories vary significantly. In Gasteropelecidae, the repeatome predominantly consists of retroelements, especially LINEs; however, in the outgroup *T. auritus*, DNA transposons are significant, constituting 13.72% of the genome and surpassing the abundance of retroelements (Table 1). These differences indicate that closely related lineages sharing homologous sex chromosome systems can evolve divergent repeat-sequence landscapes. These patterns could stem from the lineage-specific behavior of TEs, the selective retention or elimination of repeat families, and/or the varying regulatory mechanisms of hosts on particular classes of transposable elements [43,44]. However, because only one outgroup species was included, the ancestral repeatome state at their divergence cannot be reconstructed with confidence. The observed contrast could reflect lineage-specific expansion of DNA transposons in *T. auritus,* differential retention or expansion of retroelements in Gasteropelecidae, or a combination of these processes. Together, these findings indicate that the long-term conservation of a homologous sex chromosome system can occur despite substantial changes in the composition of the genomic repeat landscape.

However, while the overall TE landscape indicates genomic conservation, the satDNA fraction reveals a markedly different pattern, underscoring the dual nature of repetitive DNA evolution in Gasteropelecidae. Consistent with the view that satDNAs represent rapidly evolving components of eukaryotic genomes [45,46,47], the satellitomes of these fishes display substantial interspecific diversity and high turnover rates. This dynamic behavior is reflected in the highly variable number of satDNA families identified across the group, ranging from 59 families in *T. stellatus* to 92 in *C. strigata* (Tables S1 and S3). Despite this pronounced diversity in the number of satDNA families, our homology analyses suggest that differences among species may partly reflect differential amplification of shared ancestral sequences. Many satDNA families were detected at low abundance in other species, whereas some were not detected in every genome (Table S5). This evolutionary pattern is compatible with the “Library Hypothesis” [46,48,49], which predicts that related species inherit a common collection (or library) of satDNA sequences that independently expand or contract during evolution. In our dataset, 62% of the identified satDNA families share detectable homology and cluster into 45 groups. Notably, 12 of these homology groups include satDNAs from the outgroup *T. auritus*. Group 3 includes 22 homologous satDNA families found across all four analyzed taxa, consistent with the presence of a shared ancestral satellite lineage. These sequence-similarity patterns support a model in which quantitative shifts, rather than complete qualitative replacement, contribute substantially to satellitome diversification. Nevertheless, sequence similarity alone cannot exclude alternative explanations such as convergence or independent acquisition. As these lineages diverged, concerted evolution and unequal crossing over may have amplified specific members of the shared satellite repertoire, while other variants remained at low, nearly undetectable copy numbers. This lineage-specific reshaping of the satellitome may become particularly consequential when it occurs on sex chromosomes, where reduced recombination can facilitate the accumulation and persistence of repetitive sequences [50].

In fact, Gasteropelecidae and Triportheidae have a homologous ZZ/ZW sex chromosome system, but the W chromosome shows lineage-specific differentiation based on comparative genomic hybridization (CGH) and cytogenetic data [28]. *G. levis* was the only species for which genomic information from both sexes was available, allowing us to identify sex-biased satDNA families. Nineteen satDNA families were female-biased, with some exhibiting significant enrichment in females, such as GleSat52 (F/M = 11.10), GleSat02 (F/M = 4.74), and GleSat71 (F/M = 4.45). Female-biased abundance alone does not establish chromosomal localization and may also reflect sex-specific copy-number variation on autosomes. Nevertheless, the accumulation of satDNAs on sex chromosomes, particularly on the W and Y, has been widely documented in fishes, reptiles and primates [51,52,53,54,55]. We therefore interpret these families as candidate W-associated repeats rather than direct evidence of W localization. This interpretation gains direct cytogenetic support from *T. stellatus*, where mapping revealed pronounced W-specific accumulation of TstSat02, TstSat05, and TstSat06 (Figure 2). Thus, our data provide direct evidence for W enrichment of specific satDNAs in *T. stellatus*, while the *G. levis* data provide indirect evidence of sex-biased satellite accumulation. Together, these findings support a role for lineage-specific satDNA expansion in W chromosome differentiation but do not imply that satDNA accumulation alone initiated or drove the origin of the sex chromosome system. The combined genomic and cytogenetic patterns suggest that repetitive DNA contributes to the molecular differentiation and heterochromatinization of the W chromosome while the overall ZZ/ZW system is preserved.

## 4. Materials and Methods

### 4.1. Sampling

In this work, males and females of *T. stellatus*, *G.levis*, and *C. strigata* were investigated. Species sampling was identical to that adopted by [28], allowing direct comparisons between cytogenetic and repeatome datasets (Table 3). Genomic DNA from males of all three species was used for repeatome analyses, except for *G. levis*, for which genomic DNA from both males and females was available. Each sequencing library was derived from a single individual rather than pooled specimens. Chromosome preparations suitable for molecular cytogenetic analyses were available only for *T. stellatus.* The three studied species possess a ZZ/ZW sex-determination system [28,29,30]. All methods were carried out in accordance with relevant ethical guidelines and regulations for animal experimentation. All experimental protocols were reviewed and approved by the Animal Experimentation Ethics Committee of the Universidade Federal de São Carlos (CEUA-UFSCar; protocol no. CEUA-7994170423).

### 4.2. DNA Extraction and Genome Sequencing

Genomic DNA (gDNA) was extracted using the standard phenol-chloroform-isoamyl alcohol method [56]. Paired-end libraries of 2 × 150 bp read length were constructed from each gDNA, and low-coverage sequencing was performed on the BGISEQ-500 platform at BGI (BGI Shenzhen Corporation, Shenzhen, China). A total of 2 × 2.4 Gb of sequence data was generated for *C. strigata,* 2 × 2.3 Gb for *T. stellatus,* and 2 × 1.8 Gb and 2 × 2.7 Gb for male and female *G. levis,* respectively. All genomic sequencing reads were deposited in the NCBI Sequence Read Archive (SRA) under the following accession numbers: SRR39861389 (*Carnegiella strigata* male), SRR39861390 (*Thoracocharax stellatus* female), SRR39861391 (*Gasteropelecus levis* female), and SRR39861392 (*Gasteropelecus levis* male). In addition, publicly available sequencing datasets for *T. auritus* (SRR16764827) were incorporated into the repeatome analysis.

### 4.3. Repeatome Characterization

#### 4.3.1. Repeat Characterization by dnaPipeTE

Repeatome characterization of *Carnegiella strigata*, *Gasteropelecus levis*, *Thoracocharax stellatus*, and the outgroup *Triportheus auritus* was performed using dnaPipeTE [57] (https://github.com/clemgoub/dnaPipeTE accessed on 17 April 2026. Illumina HiSeq4000 (BGISEQ-500 platform at BGI (BGI Shenzhen Corporation, Shenzhen, China) paired-end reads were used as input for the Gasteropelecidae species, and the *T. auritus* dataset was analyzed using the same downstream repeatome workflow and classification framework. For each in-house library, a standardized number of reads (10 million of reads) was used for comparative repeatome profiling, and the datasets were subsampled to equal read counts prior to dnaPipeTE analysis. For each species, we provided its specific genome-size estimate using the genome_size parameter and used the same target coverage of 0.1× (genome_coverage 0.1). Consequently, the number of bases sampled by dnaPipeTE was proportional to the estimated genome size of each species. Two sampling/assembly iterations were conducted for each species (sample_number 2). The same RepeatMasker library, annotation threshold (RM_t 0.2), and analytical parameters were used across species. Genome size was estimated using the Animal Genome Size Database (https://www.genomesize.com/ accessed on 10 September 2026). Genome sizes of 1.36 Gb were used for *C. strigata* and *G. levis*, whereas a genome size of 1.26 Gb was used for *T. stelatus*. Because no genome-size estimate was available for *T. auritus*, a value of 1.36 Gb—the same value used for *C. strigata*—was adopted. The pipeline performs de novo assembly, annotation, and abundance estimation of repetitive elements from low-coverage genomic data. Repetitive sequences were classified using RepeatMasker against Dfam databases (version 3.6), and their genomic abundance was estimated by BLASTN-based read mapping (version 2.15). Repetitive elements were grouped into major categories, including retroelements, DNA transposons, small RNAs, simple/tandem repeats, satellites, and unclassified sequences. In the present analysis, LTR, LINE, and SINE sequences were treated as subcategories of retroelements, whereas DNA transposons represent a separate major TE class. The repeat landscape on the phylogenetic tree was constructed by converting raw sequence divergence into absolute evolutionary time using the Jukes-Cantor (JC69) correction model [58], which is a simplified special case of Kimura’s two-parameter (K2P) model [59] when transition and transversion rates are assumed to be equal, an approach widely used in comparative repeatomics. First, the raw nucleotide divergence (d) between each read and its corresponding consensus contig was calculated as d = (100 − %identity) / 100, using the values from the *reads_landscape* file generated by the dnaPipeTE pipeline. Subsequently, the corrected genetic distance (K) was calculated using the Jukes-Cantor formula: K = −3/4 × ln (1 − 4d/3), The insertion time in million years (Ma) was estimated as T = K / (2 × μ) / 10^6^, where μ represents the neutral mutation rate per site per year. We used a reference mutation rate of μ = 2.3 × 10^−9^ substitutions per site per year, following Bergeron et al. [60]. Genomic proportions based on RepeatMasker [61] annotations of contigs were calculated. All data processing and plotting were implemented in R (version 4.5.1) [62] using the packages ggplot2 (version 4.0.3) [63] and dplyr (version 1.2.1) [64].

#### 4.3.2. Satellite DNA Characterization

The satellitomes of *Carnegiella strigata*, *Gasteropelecus levis*, and *Thoracocharax stellatus* were characterized using the TAREAN tool implemented in RepeatExplorer2 [65] (accessed on 20 November 2025), which identifies satellite DNA families based on sequence cluster graph topology, together with the satMiner pipeline [11,66,67]. To generate datasets for RepeatExplorer2, raw paired-end reads were first quality-filtered using Trimmomatic, including adapter removal and quality trimming with the parameters LEADING:3, TRAILING:3, SLIDINGWINDOW:4:30, and MINLEN:101. Subsequently, a custom Python (version 2.7.18) script was used to automate the preprocessing workflow. Briefly, random subsets of 2 × 500,000 reads were generated using Seqtk, followed by the creation of interleaved FASTQ files with *shuffleSequences_fastq.pl*. Each subset was then analyzed using the RepeatExplorer2 web server with default parameters. The putative satellite DNA library was then used as a reference to remove satellite-derived reads from the corresponding trimmed dataset using DeconSeq (version 0.4.3) [68]. After filtering, a new random subset of 2 × 500,000 paired-end reads were generated and subjected to another round of analysis. This iterative process, following the SatMiner strategy, was repeated until no additional putative satellite DNA families were detected.

#### 4.3.3. Estimating the Abundance, Divergence, and Homology of Satellite DNAs

The consensus sequences obtained after the iterative SatMiner procedure were manually curated in Geneious Prime (version 2025.2.2; Biomatters Ltd., Auckland, New Zealand) [69], and the subsequent analyses were performed using scripts available in the SatMiner GitHub repository (https://github.com/fjruizruano/ngs-protocols accessed on 11 January 2026). Consensus sequences that were very similar to known multigene families, such as 18S rDNA, 5S rDNA, U1, U2, U4, U5, U6 small nuclear RNA genes, and histone H1, were compared to a reference dataset and excluded from further analyses. The remaining sequences were compiled into a final library of putative satDNA sequences, which was subsequently used for homology analyses among satellite DNA families. To assess homology among the sequences, the final satDNA consensus was analyzed using the rm_homology.py script together with the Cross_match search engine implemented in RepeatMasker (version 4.1.5) [61]. Following the criteria proposed by [11], sequences sharing >80% nucleotide identity were classified as variants of the same satDNA family, to avoid satellite library redundancy in each analysed species. For the comparative satellitome library, we grouped the satellite DNA families with a threshold of >70% to verify a higher level of similarity between the satellite DNA libraries. The genomic abundance of each satDNA family was estimated using RepeatMasker with a custom library of the identified satDNAs, and sequence divergence was calculated using the calcDivergenceFromAlign.pl script included in the RepeatMasker package [61]. To normalize satellite DNA abundance across samples, each dataset was randomly subsampled to 5 million paired-end reads (5 million reads from each mate; 2 × 150 bp). Satellite DNA abundance was expressed as the percentage of the total analyzed bases assigned to each satellite DNA family. SatDNA families were named following the nomenclature proposed by [11]. Family assignments and nomenclature standardization were performed using the satminer_quant.py pipeline, which assigned sequence variants to their respective satDNA families and generated the final family names used throughout the analyses. The complete satellitome catalogs have been deposited in GenBank under the following accession numbers: PZ793429-PZ793520 (*Carnegiella strigata*); PZ780772-PZ780855 (*Gasteropelecus levis);* and PZ793370-PZ793428 (*Thoracocharax stelatus*).

To estimate the abundance of satDNA families across species, we pooled the satDNA sequences identified in Gasteropelecidae and *T. auritus*. We then used the same short-read datasets employed in the initial abundance estimates to quantify each family in every species (Table S5).

#### 4.3.4. Primer Design, Probe Construction, and In Situ Mapping of TstSatDNAs

Out of the 59 TstSatDNA monomers identified (Table S3), the 10 most abundant satellite DNA families were initially selected for in situ mapping. Primer pairs were manually designed for TstSat01, TstSat02, TstSat04, TstSat05, TstSat06, TstSat07, TstSat08, and TstSat10 (Table S6). Primers were designed from the consensus sequence of each selected monomer using OligoCalc (https://oligocalc.eu/ accessed on 27 July 2026) and the Thermo Fisher Scientific Multiple Primer Analyzer (https://www.thermofisher.com/br/en/home/brands/thermo-scientific/molecular-biology/molecular-biology-learning-center/molecular-biology-resource-library/thermo-scientific-web-tools/multiple-primer-analyzer.html, Branchburg, NJ, USA), which were used to evaluate primer quality, including melting temperature (Tm), hairpin formation, self-dimerization, and GC content. Due to their short repeat unit lengths, TstSat02-30, TstSat06-28, TstSat07-58, and TstSat10-52 were synthesized directly with biotin molecules incorporated at the 5′ end (Sigma-Aldrich, St. Louis, MO, USA). PCR amplification was carried out with initial denaturation at 95 °C for 5 min, followed by 35 cycles of 95 °C for 60 s, 54–60 °C for 45 s, and 72 °C for 1 min 20 s, with a final extension at 72 °C for 5 min [38]. Amplification products were verified by electrophoresis on 3% agarose gels and quantified with a NanoDrop spectrophotometer (Thermo Fisher Scientific, Branchburg, NJ, USA). Probe construction was performed by nick translation with Atto488-dUTP (green) or Atto550-dUTP (red) nucleotides (Jena Biosciences, Jena, Germany) according to the manufacturer’s instructions. Probe mixtures for in situ hybridizations included 200 ng of labeled satDNA plus 50% formamide, 2 × SSC, 10% SDS, 10% dextran sulfate, and Denhardt’s buffer at pH 7.0 in a total volume of 20 µL. The FISH experiments were performed under high-stringency conditions, following the protocol described by Sassi & Cioffi [70].

### 4.4. Microscopy and Image Processing

Diploid chromosome numbers (2n) were adopted from the previously published cytogenetic studies [28,29,30]. We examined 20 metaphase spreads per individual for confirmation of the karyotypic morphology and for FISH signal assessment. The images were obtained with an Axioplan II microscope (Carl Zeiss, Jena, Germany) and processed with Image-Pro Plus 4.1 software (Media Cybernetics, Silver Spring, MD, USA).

## 5. Conclusions

Our comparative repeatomic analysis reveals that genome evolution in the studied fishes combines overall repeatome stability with rapid satDNA diversification. Despite substantial evolutionary divergence, the three genera retain broadly conserved repeat landscapes dominated by retroelements, while their satellitomes have undergone pronounced lineage-specific changes consistent with differential amplification of shared satellite sequences. This dynamic is particularly evident in the conserved ZZ/ZW sex chromosome system, where female-biased and W-enriched satDNAs show that substantial molecular differentiation can coexist with long-term conservation of sex chromosome architecture. Together, these findings show that repetitive DNA does not evolve as a homogeneous genomic component but rather through distinct evolutionary trajectories, with satellite DNAs contributing to chromosome and sex chromosome differentiation. Gasteropelecidae therefore provides a compelling model for understanding how stable chromosome systems can accommodate extensive sequence-level genome diversification over evolutionary timescales. A major next step will be the generation of chromosome-level reference genomes for these species, providing a broader and more comprehensive view of repetitive DNA landscapes and revealing how major repeat classes are organized and distributed across individual chromosomes.

## Figures and Tables

**Table 1:** Genome-wide composition of repetitive DNAs in three Gasteropelecidae species and the outgroup *Triportheus auritus*.

Category	Type	*Carnegiella strigata*	*Gasteropelecus levis*—Female	*Gasteropelecus levis*—Male	*Thoracocharax stellatus*	*Triportheus auritus*
**Retrotransposons (Class I)**	LTR	2.33%	1.44%	1.03%	1.69%	0.73%
	LINE	5.91%	5.99%	5.88%	5.20%	3.06%%
	SINE	1.49%	0.86%	0,94%	0.24%	0.73%
						
**DNA transposons (Class II)**	DNA transposons	7.57%	6.78%	5.36%	5.09%	13.72%
	Helitron	0.39%	0.38%	0.29%	0.03%	0.26%
						
**Tandem repeats**	rRNA	0.48%	0.41%	0.55%	0.69%	0.52%
	Low Complexity	0.27%	0.16%	0.21%	0.06%	0.03%
	Satellites	0.11%	0.74%	0.27%	0.08%	0.09%
	Simple Repeat	1.44%	2.33%	1.34%	2.32%	0.98%
**Other elements**		0.59%	0.41%	0.23%	0.62%	0.38%
**Unannotated**		11.97%	8.77%	9.10%	8.51%	12.10%
**Total repeat content**		32.75%	28.26%	25.19%	24.51%	32.60%

**Table 2:** Homology groups identified in the satellitomes of Gasteropelecidae species and *Triportheus auritus,* based on the sequence homology analysis presented in Table S4. The table summarizes satDNA families assigned to the same homology groups and exhibiting sequence similarities greater than 70%. Similarity values represent the range of pairwise sequence similarities observed among the satDNA families within each homology group.

Group	*Triportheus* *auritus*	*Gasteropelecus* *levis*	*Carnegiella* *strigata*	*Thoracocharax* *stellatus*	Similarity *(%)*
3		GleSat47-1401		TstSat22-1302	71
5		GleSat06-84	CstSat19-82		78
9			CstSat10-58	TstSat07-58	98
12	TauSat20-73	GleSat20-73	CstSat13-73	TstSat17-73	95–96
13		GleSat09-1596	CstSat14-1526		74
14		GleSat18-21	CstSat15-21		100
17	TauSat06-42	GleSat27-39	CstSat18-40		80–95
23		GleSat61-29	CstSat26-29	TstSat34-29	86–90
24		GleSat24-67	CstSat27-67	TstSat43-67	96–97
25		GleSat40-58	CstSat28-58	TstSat46-59	86–92
26	TauSat19-76	GleSat36-73	CstSat30-73	TstSat29-73	87–100
27			CstSat31-58	TstSat42-52	88
30		GleSat31-29	CstSat35-29	TstSat28-29	93–100
31		GleSat30-30	CstSat36-30	TstSat30-30	100
33		GleSat37-59	CstSat38-65		83
34		GleSat08-33	CstSat40-33		87
35	TauSat26-82	GleSat32-72	CstSat42-82	TstSat39-82	87–98
38		GleSat29-29	CstSat45-29	TstSat21-29	97–100
40		GleSat70-62	CstSat47-62		89
43	TauSat46-67	GleSat53-67	CstSat50-67		88–99
46		GleSat50-51	CstSat53-51	TstSat51-51	94–98
53	TauSat12-66	GleSat64-68	CstSat62-68		92–100
55		GleSat44-33	CstSat64-32		94
60		GleSat39-58	CstSat72-58		95
65			CstSat78-153	TstSat49-153	78
67		GleSat55-68	CstSat82-67		88
72		GleSat74-46	CstSat88-49		90
79		GleSat03-52		TstSat10-52	88
81		GleSat05-28		TstSat06-28	96
91	TauSat16-29	GleSat33-29			100

**Table 3:** Sampling locations in Brazil and sample sizes for the analyzed species.

Species	Sample Site	Coordinates	N
*Gasteropelecus levis*	Manaus (AM)	2°49′02.7″ S; 60°30′38.0″ W	21 ♀; 19 ♂
*Carnegiella strigata*	Manaus (AM)	2°35′42.9″ S; 60°02′23.8″ W	24 ♀; 18 ♂
*Thoracocharax stellatus*	Barra do Bugres (MT)	14°47′25.7″ S; 57°17′40.2″ W	31 ♀; 20 ♂

*MT = Mato Grosso; AM = Amazonas*.

## Data Availability

The raw sequencing reads generated in this study were deposited in the Sequence Read Archive (SRA-NCBI) under the accession numbers SRR39861389 (*Carnegiella strigata* male), SRR39861390 (*Thoracocharax stellatus* female), SRR39861391 (*Gasteropelecus levis* female), and SRR39861392 (*Gasteropelecus levis* male). The satellite DNA catalogs generated for *C. strigata*, *G. levis*, and *T. stellatus* are available in GenBank under accession numbers PZ793429–PZ793520, PZ780772–PZ780855, and PZ793370–PZ793428, respectively. In addition, the publicly available sequencing dataset for *Triportheus auritus* (SRR16764827) was incorporated into the repeatome analysis.

## Supplementary Materials

The following supporting information can be downloaded at https://www.mdpi.com/article/10.3390/ijms27198720/s1.

## Abbreviations

The following abbreviations are used in this manuscript:

2n	Diploid number
BLASTN	Basic Local Alignment Search Tool for Nucleotides
CGH	Comparative Genomic Hybridization
FISH	Fluorescence In Situ Hybridization
K2P	Kimura two-parameter model
LINEs	Long Interspersed Nuclear Elements
LTR	Long Terminal Repeat
Ma	Million years
NCBI	National Center for Biotechnology Information
PCR	Polymerase Chain Reaction
RUL	Repeat Unit Length
SDS	Sodium dodecyl sulfate
SINEs	Short Interspersed Nuclear Elements
SSC	Saline–Sodium Citrate
TAREAN	Tandem Repeat Analyzer
TEs	Transposable Elements
Tm	Melting temperature
